# Comparison of long-pulsed alexandrite laser and topical tretinoin-ammonium lactate in axillary acanthosis nigricans: A case series of patients in a before-after trial

**Published:** 2016

**Authors:** Amirhoushang Ehsani, Pedram Noormohammadpour, Azadeh Goodarzi, Mostafa Mirshams Shahshahani, Seyede Pardis Hejazi, Elhamsadat Hosseini, Arghavan Azizpour

**Affiliations:** 1 Department of Dermatology, Razi Hospital, Tehran University of Medical Sciences (TUMS), Tehran, Iran.; 2 Department of Dermatology, Rasul Akram Hospital, Iran University of Medical Sciences (IUMS), Tehran, Iran.; 3 Baghiatallah University of Medical Sciences, Tehran, Iran.

**Keywords:** Acanthosis nigricans, Long-pulsed alexadrite laser, Tretinoin, Ammonium Lactate

## Abstract

**Background:**

Acanthosis nigricans (AN) is a brown to black, velvety hyperpigmentation of the skin that usually involves cutaneous folds. Treatment of AN is important regarding cosmetic reasons and various therapeutic modalities have been used for these purposes. The goal of this study was to compare the effectiveness of long-pulsed alexandrite laser and topical tretinoin-ammonium lactate for treatment of axillary-AN.

**Methods:**

Fifteen patients with bilateral axillary-AN were studied in Razi Hospital, Tehran, Iran. Diagnosis was confirmed by two independent dermatologists. Each side skin lesion was randomly allocated to either topical mixed cream of tretinoin 0.05%- ammonium lactate 12% or long-pulsed alexandrite laser. Duration of treatment was 14 weeks. At endpoint, the mean percent reduction from baseline in pigmentation area was compared between the two groups.

**Results:**

The study population consisted of 15 patients three males and 12, females. The mean age of patients was 28.5±4.9 years. The mean percent reduction was 18.3±10.6%, in tretinoin/ammonium lactate group and 25.7±11.8% in laser group (P=0.004).

**Conclusion:**

These findings indicate that the application of alexandrite laser is a relative effective method for treatment of axillary-AN. However, this issue requires further studies with prolonged follow-up period.

Acanthosis nigricans (A.N) is a cutaneous disorder characterized by brown to black, poorly circumscribed, velvety hyperpigmentation that is sometimes associated with hyperkeratotic plaques. It is usually found in cutaneous folds, such as posterior and lateral folds of the neck and the axilla (1-2). The exact prevalence of acanthosis nigricans is unknown but in few studies it has been estimated up to 7% in normal population (3). Acanthosis nigricans is much more common in people with darker skin, so that in whites, the prevalence is less than 1% (4-5). Regarding histopathologic aspect, A.N is characterized by the thickening of the stratum corneum, ancanthosis, papillomatopsis, and minimal dermal involvement. Thickness of acanthosis is variable and usually minimal. In acanthosis nigricans, hyperkeratosis is a more common reason of dark color than the increased pigmentation. Sometimes in the lesion of A.N, there is secretion accumulation of lymphocytes, plasma cells and neutrophils, which can lead to horn pseudocyst formation. 

Acanthosis nigricans can be associated with different kinds of syndromes, such as insulin resistance and mutation in fibroblast growth factor receptor. Although uncommon, but acanthosis nigricans may be induced by several medications such as corticosteroids, niacin, insulin, oral contraceptives and protease inhibitors (6-8). The goal of therapy in acanthosis nigricans is to correct the underlying disorder and treatment of lesion of acanthosis nigricans mainly is due to cosmetic reasons. Topical medications with some therapeutic success have been used for acanthosis nigricans, including: topical retinoids and lactates (9), topical vitamin A (10), and topical keratolytics (11). Cryotherapy is another method treatment in acanthosis nigricans using liquid nitrogen, and its efficacy depends on several factors, including; depth of the lesion, amount of lesions vascularity, duration of each freezing cycle and number of performed freezing cycles. The side effects most commonly encountered with this type of treatment are hypopigmentation and local recurrences (12). Curettage, simple excision, electrodessication and dermabrasion are the other treatment modalities. 

Pulsed dye laser (PDL) showed low efficacy in the treatment of acanthosis nigricans due to its low energy and low penetration of laser beams. Alexandrite laser with 755 nm wave length can target melanin pigments in keratinocytes; therefore, it is capable to destruct melanin containing keratinocytes. Alexandrite laser has a high penetration rate and able to destroy AN lesions without any damage to surrounding tissues (13-15). The efficacy of long-pulsed alexandrite laser has been demonstrated for the treatment of many pigmented lesions including axillary-acanthosis nigricans until now, although there is a high prevalence of acanthosis nigricans and its interfering cosmetic problems, no treatment of any choice has been proposed with this regard. We decided to evaluate the efficacy of alexandrite laser in the treatment of acanthosis nigricans and compare its cosmetic results with other routine topical peeling agent which was a mixed cream of tretinoin and ammonium lactate. 

## Methods

The study population comprised 15 cases of AN which was conducted in Razi Hospital, Tehran, Iran in 2012. This was an assessor and analyst-blinded, randomized, controlled before-after clinical trial study. The study participants were recruited among these subjects with AN in the axillary regions. The diagnosis was confirmed clinically by two dermatologists.

Criteria for inclusion were: presence of bilateral axillary- acanthosis nigricans , normal laboratory test regarding blood sugar, hormonal profile and liver function tests, no history of any medication consumption which was considered as drug-related cause of acanthosis nigricans such as nicotinic acid, fusidic acid and oral contraceptives, no underlying malignancy, not pregnant, no skin disorders localized to the treated area such as vitiligo (due to Koebner phenomenon), no history of taking oral retinoids in the last six months (due to contra-indication in laser therapy). Based on an earlier study (16-18), a 20% or greater improvement in skin lesions compared to baseline values was considered as treatment response. With regard to type I error of 5% and the study power of 80%, the minimum sample size required in each group was 15 patients. 

According to the study design, one side axillary-acanthosis nigricans was treated with laser while the contra lateral side with topical tretinoin 0.05%/ammonium lactate 12%. After the ethics approval of the committee, 15 patients were enrolled into the study after explaining the complete information about the aims of the study, likewise filling out the informed consent form, sequencing performed-based on balanced block randomization, as well as evaluating 15 binary sites (right and left side for each patient, totally 30 sites). Each side of axilary region was allocated randomly to laser therapy or treatment with tretinoin 0.05%/ ammoniumlactate 12%.

Combination of tretinoin 0.05% and ammonium lactate 12% was used every other night, and long-pulsed alexandrite laser for the opposite side was repeated every 3 weeks. Four laser shot with 18 mm spot size and energy of 20 mJ also DCD off as a 2×2 cm^2, ^were considered for therapy. Changes in response to treatment were determined by the comparison of photographs taken at baseline and treatment duration for each patient was 14 weeks, and each patient received 5 sessions of laser with 3 weeks interval. At the end of this therapy course and after 1 week interval (for relieving the local skin reactions and irritations), both side lesions were photographed again and was assessed by a blinded expert dermatologist (the assessor of study who did not know the type of intervention used for each side axillary-acanthosis nigricans). The assessor determined the pigmentation reduction percent after therapy, based on visual analogue scale (VAS). The data were finally analyzed by a blind researcher. 

For data analysis, SPSS software Version 22 was used. Descriptive tests (such as mean, median and standard deviation) were performed for qualitative variables and analytic tests (such as t-test, ANOVA) for quantitative ones. A p-value under 0.05 was considered as statistically significant.

## Results

Fifteen patients including 3 men and 12 women were enrolled into the study. The mean age of the patients was 28.5±4.9 years ranging from 18 to 37 years .The mean percent reduction in the pigmentation after laser therapy was 25.67±11.78 (ranging from 15 to 50 percent) and 18.33±10.63 (ranging from 10 to 50 percent). by topical therapy. The difference was statistically different (P=0.004) (table 1).

**Table 1:** Mean amount of pigmentation reduction in two treatment groups of axillary-acanthosis nigricans

**Treatment method**	**Mean reduction in pigmentation (%)**	**Least amount of decrease** **in pigmentation(%)**	**Maximum amount of ** **decrease in pigmentation(%)**	**P-value**
Laser therapy	25.67±11.78	15	50	0.004
Topical tretinoin 0.05% ammonium lactate 12%	18.33±10.63	10	50	

## Discussion

The findings of this study indicated significantly higher efficacy of laser therapy versus topical treatment of AN. Pigmented cutaneous lesions form a major part of problems encountered in skin-related disorders. Among the pigmented lesions, acanthosis nigricans is somehow unique due to needing to long-term therapies with peeling agents and also topical retinoids. But the treatment results are not generally satisfactory. With regard to the high prevalence of acanthosis nigricans, particularly in overweight patients and lack of any effective treatments, identification of new treatment modalities seems to be necessary and worthwhile (1-5). According to this study, the mean age of the patients was less than 30 years old. In this age group, the psychological burden of acanthosis negricans on quality of life is important. 

Although none of the treatment modality resulted in complete recovery of skin lesion, but reduction in pigmentation was significantly higher in the alexandrite laser treatment group rather than the topical tertinoin/ammonium lactate group. The efficacy of long-pulsed alexandrite laser has been shown also in other similar studies, for example, Rosenbach in 2002 found that alexandrite laser had acceptable results in the treatment of acanthosis nigricans with low level of therapy-related complications (16). In addition, this author in another study proved a 95% improvement in acanthosis nigricans lesions after 7 sessions of laser therapy without recurrences in a 2 -year follow-up (17).

In conclusion, the results of this study indicates that using long-pulsed alexandrite laser can effectively and safely treat axillary-acanthosis nigricans, which exerts greater beneficial effect than routine topical peeling agents. The results of this study should be considered with limitations as regard low sample size. In addition, in the present study, the mean changes in pigmentation area was considered as the main variable, whereas, further treatment is expected to influence on the thickness of lesions as well as the total cosmetic result. Long term treatment and follow-up is expected to show greater efficacy. This context requires further studies.

## Notes

**Funding: **This study was ethically and scientifically approved and then financially supported by a research grant received from Tehran University of Medical Sciences, Tehran, Iran.
